# Differential pericyte pathology in the human retina and brain in diabetes mellitus and Alzheimer’s disease

**DOI:** 10.3389/fnins.2026.1749112

**Published:** 2026-02-05

**Authors:** Noëlle Bakker-van Bugnum, Aïcha A. Croes, Eva Prevaes, Cornelis J. F. van Noorden, Reinier O. Schlingemann, Ingeborg Klaassen

**Affiliations:** 1 Department of Ophthalmology, Ocular Angiogenesis Group, Amsterdam UMC Location University of Amsterdam, Amsterdam, Netherlands; 2 Amsterdam Cardiovascular Sciences, Microcirculation, Amsterdam, Netherlands; 3 Amsterdam Neuroscience, Cellular and Molecular Mechanisms, Amsterdam, Netherlands

**Keywords:** Alzheimer’s disease, brain, diabetes, diabetic retinopathy, neurovascular unit, pericyte pathology, retinal disease

## Abstract

**Introduction:**

In diabetic retinopathy, pericyte dysfunction, pericyte loss, and inner blood–retinal barrier (iBRB) dysfunction contribute to neurovascular unit (NVU) impairment. Diabetes mellitus (DM) is also associated with increased risk of Alzheimer’s disease (AD), and it has been hypothesized that DM-induced NVU impairment in brain capillaries, including pericyte dysfunction, may contribute to AD pathogenesis. In the present hypothesis-generating explorative study, we investigated pericyte characteristics in the iBRB in patients with type 2 DM with or without diabetic retinopathy (DR), and in the blood–brain barrier (BBB) in type 2 DM and AD.

**Methods:**

We analysed human retina and brain samples from controls and donors with DM and/or AD. Immunofluorescence staining for NG2, PDGFRβ, and αSMA was performed to analyse pericyte marker expression, vascular staining coverage, and pericyte cellular density on capillaries.

**Results:**

In control retina and brain, the average pericyte staining coverage of capillaries was 70–80% based on NG2 and PDGFRβ expression, but only 25% when based on αSMA expression. Pericyte densities were 7 and 9 pericytes/mm capillary length in the control retina and brain, respectively. DM and DR retinas showed marked density reductions to 4 pericytes/mm capillary length. In DM without DR, retinal vascular staining coverage of NG2 and PDGFRβ decreased to 50–56%. In DR retinas, vascular coverage based on NG2 staining was comparable to controls, whereas coverage based on PDGFRβ staining was significantly reduced to 45%. Such reductions were not observed in brain samples from donors with DM or AD; however, NG2 staining was reduced in all patient groups. Both NG2 and PDGFRβ staining were markedly reduced in brain samples from donors with both DM and AD.

**Discussion:**

These trends suggest a specific pericyte pathology in the brain in cases of DM and AD, particularly in patients with both conditions, which differs from the well-characterized pericyte loss observed in the diabetic retina.

## 1 Introduction

Individuals with diabetes mellitus (DM) carry a higher risk of developing dementia, but the mechanisms underlying this association remain unclear (Little et al., 2022). Diabetes impairs the retinal neurovascular unit (NVU) through various molecular and cellular alterations, driving diabetic retinopathy (DR) progression (Bosma et al., 2019). Patients with diabetes may exhibit thickening of the basal lamina, decreased junctional protein levels, pericyte dysfunction and loss, neurodegeneration, increased endothelial transcellular transport, microglia activation, glial cell dysfunction and loss, and endothelial cell damage or death. It is presently unknown whether similar NVU changes occur in the brain in cases of DM or DM-associated Alzheimer’s disease (AD). Studies in experimental animal models of diabetes, and incidental reports on human DM brain tissue, have yielded conflicting results regarding diabetes-induced NVU impairment (Dai et al., 2002; Trost et al., 2016; Bogush et al., 2017; Ogura et al., 2017; Park et al., 2017; Villasenor et al., 2017; Santos et al., 2018; Little et al., 2023; Feng and Gao, 2024).

Pericytes play crucial roles in the formation and maintenance of the inner blood–retinal barrier (iBRB) and blood–brain barrier (BBB) (Klaassen et al., 2013; Klaassen et al., 2015; Trost et al., 2016; Little et al., 2022; van Noorden et al., 2024). Retinal pericyte loss is an early event in diabetic retinopathy (Klaassen et al., 2013; Bosma et al., 2019; Little et al., 2022), which has also been reported to occur in the brain during experimental diabetes (El-Ghazawi et al., 2024; Feng and Gao, 2024), although human studies are lacking. Pericyte loss occurs via migration away from capillaries or cell death (Klaassen et al., 2013), and may be caused by inflammatory mechanisms, oxidative stress, increased levels of advanced glycation end-products, high levels of angiopoietin-2, and thickening of the basal lamina (Bosma et al., 2019; Little et al., 2022; Li and Fan, 2023). Pericyte loss can result in changes in blood flow, microaneurysm formation, abnormal capillary remodeling and nonperfusion, and increased capillary permeability resulting in edema (Warmke et al., 2016; Little et al., 2022; Benarroch, 2023).

No clinical biomarkers are currently available to identify DM patients at higher risk of cognitive decline or dementia (Ehtewish et al., 2022; Little et al., 2022). Given the shared embryonic origin of the retina and brain (Trost et al., 2016), it is possible that retinal biomarkers reflecting NVU degeneration could serve as early indicators of cognitive impairment in DM patients. However, this assumes that common vascular pathological mechanisms underlie both brain and retinal changes in DM and AD. To further explore this possibility, in the present study, we investigated the brain and retinal vasculature in DM and AD, with particular focus on the pericytes of the NVU in the human retina and brain.

In this explorative hypothesis-generating study, we examined pericyte alterations in the iBRB in DM, and in the BBB in DM and AD. To characterize pericytes in human retina and brain tissues, we performed immunofluorescence staining for neural/glial antigen 2 (NG2), platelet-derived growth factor receptor beta (PDGFRβ), and alpha smooth muscle actin (αSMA). Apart from our previous investigations (Kuiper et al., 2004; Bakker et al., 2025), no studies have systemically analyzed NG2, PDGFRβ, and αSMA expression in pericytes of the adult human retina and brain. NG2 and PDGFRβ are receptors on the pericyte membrane; upon their activation, intracellular signaling pathways facilitate pericyte proliferation, and pericyte recruitment to newly formed vessels (Trost et al., 2016; van der Wijk et al., 2018; Bakker et al., 2025). Interactions with endothelial cells enable maturation of the iBRB and BBB (Klaassen et al., 2013). αSMA is a contractile protein found in smooth muscle cells and pericytes, which likely mediates pericyte contraction to regulate capillary diameter and blood flow in response to neural activity (Alarcon-Martinez et al., 2018; Girolamo et al., 2022). Our analyses included retinal samples from donors with type 2 DM, with or without DR, and rare brain tissues from donors with type 2 DM, AD, or both conditions. This comprehensive approach enabled quantitative comparisons of pericyte cellular density and vascular staining coverage based on these pericyte markers, as well as assessment of the marker expression patterns in neurovascular pathological conditions affecting both the retina and brain.

## 2 Materials and methods

### 2.1 Retinal tissue

Human post-mortem retina tissue samples were processed as previously described (Bakker et al., 2025). Retinal tissues were obtained from three donor groups: donors with type 2 DM without DR (mean age of 67 ± 2.3 years), donors with type 2 DM and DR (mean age of 65 ± 8.1 years), and non-diabetic controls (mean age of 67 ± 0.8 years) (*n* = 4 for all groups). The male-to-female ratio was 2:2 (DM no DR), 1:3 (DR), and 1:3 (controls). Table 1 summarizes the donors’ characteristics. The current research performed on human eyes is in accordance with all requirements stated in the Dutch law “Wet op orgaandonatie” that describes the use of donor material for research purposes. According to this law, donors provide written informed consent for donation with an opt out of left-over material for related scientific research purposes. Specific requirements for the use for scientific research of left-over material originating from corneal grafting have been described in an additional document formulated by the Ministry of Health, Welfare, and Sport and the Bio Implant Services (BIS) Foundation (Eurotransplant; Leiden, the Netherlands, July 21, 1995; 6,714.ht). The eyes were stored anonymously and, therefore, approval of their use by the Ethics Committee was not required by Dutch law.

**Table 1:** Information about retinal tissue donors.

Case	Laser yes/no	Sex (F/M)	Age at death (y)	Post-mortem delay (h)	Age at diabetes onset (y)	Insulin use (yes/no)	Diagnosis	Cause of death
Control								
1	No	F	68	14		No	Stomach cancer	Cancer (stomach)
2	No	F	68	17		No	Myocardial infarction	Myocardial infarction
3	No	F	67	12		No	Atherosclerotic disease, chronic kidney failure, narrowed coronary arteries	Atherosclerotic disease
4	No	M	66	9		No	Lung embolism	Melanoma
Mean ± SD			67 ± 0.8	13.0 ± 2.9				
DM no DR								
5	No	M	67	15	Unknown	No	Vascular disease, leg amputation	Aortic aneurysm
6	No	M	65	11	63	No	COPD, acute bronchitis, hyperthyroidism	Lung condition
7	No	F	66	13	Unknown	No	Brain metastasis	Brain metastasis
8	No	F	71	10	Unknown	No	Breast carcinoma, alcohol abuse, hepatocellular carcinoma	Hepatic coma, liver failure
Mean ± SD			67 ± 2.3	12.3 ± 1.9				
DM + DR								
9	Yes	F	66	19	Unknown	No	Heart failure	Heart failure
10	Yes	F	61	13	<49	Yes	Heart failure	Heart failure
11	Yes	M	77	18	62	Yes	Heart failure, kidney stones, hypertension, cerebral hematoma	Heart failure
12	Unknown	F	55	10	41	No	Hypertension, adiposity, high cholesterol, lipid disorder, fatty liver	Heart failure
Mean ± SD			65 ± 8.1	15.0 ± 3.7				

DM, diabetes mellitus; DR, diabetic retinopathy.

### 2.2 Immunofluorescence staining of retinal tissue

Human retinal flatmounts were subjected to immunofluorescence double staining for NG2 and PDGFRβ, and for αSMA and PDGFRβ, which was performed after optimization, as recently described (Bakker et al., 2025). Retinal flatmounts were used instead of tissue sections, to enable 3D reconstruction of pericytes on capillaries and of their distribution across the vascular network, including their position relative to arteries, veins, and microvascular branches. Table 2 lists the primary antibodies used in this study. Flatmounts stained for laminin, NG2, and PDGFRβ were incubated with the following secondary antibodies: donkey anti-goat Cy3, donkey anti-rabbit Alexa Fluor™ 488, and donkey anti-mouse Alexa Fluor™ 647 (Table 3). Flatmounts stained for laminin, αSMA, and PDGFRβ were incubated with the following secondary antibodies: donkey anti-goat Cy3, donkey anti-rabbit Alexa Fluor™ 488, and donkey anti-mouse Alexa Fluor™ 647 (Table 3).

**Table 2:** Primary antibodies used in this study.

Antigen	Host species	Source	Working dilution
Laminin	Rabbit	Abcam, Ab11575	1:1,000
NG2	Mouse	Merck Life Science, MAB2029	1:100 (retina)/1:200 (brain)
PDGFRβ	Goat	R&D Systems, AF385	1:100
αSMA	Mouse	DAKO, M0851	1:500

R&D Systems (Minneapolis, MI); DAKO (Santa Clara, CA).

**Table 3:** Secondary antibodies used in this study.

Antibody	Conjugate	Source	Working dilution
Donkey anti-rabbit IgG	Alexa Fluor™ 488	Invitrogen, A-21206	1:400
Donkey anti-mouse IgG	Alexa Fluor™ 488 plus	Invitrogen, A-32766	1:1,000
Donkey anti-goat IgG	Cy3	Jackson, 705–165-147	1:200
Donkey anti-rabbit IgG	Alexa Fluor™ 647	Invitrogen, A-31573	1:500
Donkey anti-mouse IgG	Alexa Fluor™ 647	Invitrogen, A-31571	1:200

Invitrogen (Waltcam, MA); Jackson ImmunoResearch Laboratories (West Grove, PA).

### 2.3 Brain tissue

Post-mortem samples of the human frontal cortex were provided by the Netherlands Brain Bank, Amsterdam, The Netherlands (NBB project 1345S). Frontal cortex samples were selected, because pathological features, including vascular pathology (Thomas et al., 2015; Govindpani et al., 2019), appear in the frontal cortex during early stages of dementia (Beason-Held et al., 2013; DeTure and Dickson, 2019; Metaxas et al., 2019). All donors or their relatives provided written informed consent for brain autopsy and the use of brain tissue for research purposes. This usage was in accordance with the Declaration of Helsinki on the use of human material for scientific research. Brain tissues were collected within several hours post-mortem, dissected, snap frozen in liquid nitrogen, and stored at −80 °C until sectioning. Donors were classified into four groups: donors with type 2 DM (mean age of 72 ± 9.6 years) (*n* = 4), donors with AD (mean age of 74 ± 7.6 years) (*n* = 4), donors with type 2 DM and AD (mean age of 74 ± 6.6 years) (*n* = 4), and non-diabetic non-AD controls (mean age of 70 ± 12.2 years) (*n* = 3). Clinical diagnoses were confirmed by autopsy findings. The male-to-female ratio was 2:2 (DM), 3:1 (AD), 3:1 (DM + AD), and 1:2 (controls). Table 4 summarizes the donors’ characteristics. The subject groups were age-matched to the controls, to account for age-dependent loss of BBB integrity (Michalicova et al., 2020).

**Table 4:** Information about brain tissue donors.

Case	Sex (F/M)	Age at death (y)	Post-mortem delay (h)	Age at disease onset (y)	Braak stage (0–6)	Vascular pathology in frontal cortex	Neuropathology in frontal cortex	Cause of death
Control								
1	F	66	6.3		1	Slight atherosclerosis	Myelin pallor, only MS plaques, no AD plaques	Cancer
2	M	58	7.7		0	Slight atherosclerosis	Partly absent myelination, no amyloid plaques	Myocardial ischemia
3	F	87	4.6		2	Slight atherosclerosis	No CAA, moderate number of amyloid diffuse plaques, a few classic plaques, no tangles	Pneumonia
Mean ± SD		70 ± 12.2	6.2 ± 1.3					
DM								
4	F	70	6.0	50	2	Slight atherosclerosis, perivascular edema, some iron pigment	Few diffuse plaques, no CAA, no tangles	Kidney failure
5	M	67	9.0	61	1	Slight atherosclerosis	No CAA, moderate-to-large number of diffuse plaques, no tangles, slight perivascular edema	Aortic aneurysm, asystole
6	M	65	7.2	Unknown	2	Slight atherosclerosis	No plaques or tangles, some tau-positive cells	Fever, neuroleptic malignant syndrome
7	F	86	7.5	Unknown	2	Slight atherosclerosis, severe perivascular edema	No CAA; moderate number of plaques, equal numbers of diffuse and classic types; no tangles	Cancer
Mean ± SD		72 ± 9.6	7.4 ± 1.2					
AD								
8	F	78	7.5	71	5	Slight atherosclerosis	Moderate CAA; no dyshoric angiopathy; no striking capillary angiopathy; moderate-to-many amyloid plaques, especially diffuse; many tangles	Dehydration
9	M	75	6.3	66	5	Slight atherosclerosis	Slight CAA; many diffuse and classic plaques; presence of tangles, neuritic plaques, and Lewy bodies	Cachexia
10	M	63	9.8	56	6	Slight to severe atherosclerosis	Slight CAA with dyshoric angiopathy and no capillary angiopathy; many plaques, diffuse types and classic types; many tangles	Dehydration/cachexia
11	M	80	5.5	79	6	No atherosclerosis	CAA present, many diffuse plaques and few classic plaques, presence of tangles and Lewy bodies	Dehydration and pneumonia
Mean ± SD		74 ± 7.6	7.3 ± 1.9					
DM + AD								
12	M	67	5.4	DM and Dem: unknown	5	Slight atherosclerosis	Many senile plaques, neurofibrillary tangles and many neuropil threads; moderate number of plaques, mainly “classic” plaques with large cores	Cerebral infarction
13	M	71	4.0	DM: 57; Dem: 64	6	Slight atherosclerosis	Large plaques with coarse fibrils of weakly staining amyloid, few diffuse plaques, many neuritic plaques, and a moderate amount of tangles	Dementia, delirium, and dehydration
14	M	82	8.5	DM: unknown; Dem: 80	5	Perivascular oedema	Many amyloid beta depositions, including diffuse plaques, classic plaques, and small depositions; many neuropil threads, tangles, and dispersed neuritic plaques; myelination is normal	Heart failure
15	F	77	3.8	DM: 73; Dem: 73	5	Moderate atherosclerosis	Slight to moderate CAA, mostly diffuse plaques, few-to-moderate neuritic plaques, moderate number of tangles, many Lewy inclusions, many Lewy threads	Infection and dehydration
Mean ± SD		74 ± 6.6	5.4 ± 2.2					

AD, Alzheimer’s disease; CAA, cerebral amyloid angiopathy; DM, diabetes mellitus; Dem, dementia.

### 2.4 Immunofluorescence staining of brain tissue

Brain tissue specimens were cut into 20-μm-thick sections at −20 °C, using an Microm Cryo Star HM 560 cryostat (Thermo Fisher Scientific), which were stored at −80 °C until further use. These thick brain sections enabled 3D visualization of pericytes. In preparation for immunofluorescence staining, the tissue sections were air dried at room temperature (RT) for 20 min. Next, the sections were fixed with 4% formaldehyde (28,908; Thermo Fisher Scientific) for 20 min, and then washed once in 3 × PBS for 10 min. Non-specific fluorescence was blocked following an adaptation of the methods described by Ma et al. (2018). The sections were blocked and permeabilized by incubation in PBS supplemented with 0.2% BSA, 0.3% Triton-X (T-X), and 5% normal donkey serum, at RT for 1 h. Directly after incubation with blocking buffer, the sections were incubated with primary antibodies (Table 2) diluted in 0.2% BSA in PBS, overnight at 4 °C. Subsequently, the sections were washed three times with PBS for 10 min each. To reduce lipofuscin autofluorescence, the sections were incubated in Trueblack (Zhang et al., 2022; Stillman et al., 2023) (23,007; Biotium, Fremont, CA) diluted 20 × in 70% ethanol for 30 s, and were then washed three times in PBS for 10 min each. Next, the sections were incubated for 1 h at RT in the dark with the following secondary antibodies: donkey anti-mouse Alexa Fluor™ plus 488, donkey anti-goat Cy3, and donkey anti-rabbit Alexa Fluor™ 647 (Table 3). Following this incubation, the sections were washed three times with PBS for 10 min each. Finally, the sections were mounted with Vectashield antifading mounting medium containing DAPI (H-1200-10; Vector Laboratories), covered with a cover glass, and sealed with transparent nail varnish.

### 2.5 Microscopical analysis of pericyte staining in 3D

Imaging was performed as previously described (Bakker et al., 2025). Images of at least 10 randomly selected areas in a section were analyzed for quantification for each patient group (*n* = 3–4 donors per group). For each marker, the exposure time and laser intensity were maintained constant for each section, among all patient groups. Three-dimensional confocal images were projected with maximum intensity projection from 5.89-μm-thick z-stacks for the retinal flatmounts, and from 4.5-μm-thick z-stacks for brain samples, each with a step size of 0.346 μm.

### 2.6 Quantification

Pericyte staining was quantified using ImageJ software (National Institutes of Health). Relative vascular staining coverage was calculated as % positive pericyte marker area/total vessel area, as determined by laminin immunofluorescence staining. Regions of interest (ROIs) were drawn manually (brain samples) or automatically (retina samples) around the vasculature, using ImageJ running macro script 1. Details are provided in Supplementary Figure 1. To select for capillaries and avoid small fragments of blood vessels, respectively, we excluded blood vessels with a diameter of >10 μm and those with a length of <20 μm.

Before quantifying vascular staining coverage, we determined the background intensity for each pericyte marker in three equally-sized square regions, outside of blood vessels, in every image. The mean intensity from these background regions was used as the threshold for background subtraction using the ImageJ macro script 2 (Supplementary Figure 2).

Vascular staining coverage was quantified as the percentage of pericyte area colocalized with vascular ROI, as determined by laminin immunofluorescence staining, using ImageJ Area Fraction measurement. The results were averaged across all images per donor.

Pericyte cellular density was quantified by manually counting the NG2-positive pericyte bodies (identified based on their characteristic nodular or bump-shaped morphology) per mm of capillary length. To obtain vascular area measurements, laminin-positive blood vessels were traced in ImageJ, generating ROIs for each image. To measure capillary length in the laminin channel, a line was manually drawn through the central axis of each blood vessel in ImageJ. Finally, pericyte cellular density was calculated as the average number of pericyte bodies per mm capillary length for each donor.

### 2.7 Statistical analysis

Statistical tests were performed using GraphPad Prism v10 (GraphPad Software, La Jolla, United States). A Kruskal-Wallis test was used for comparison between patient groups (*n* = 4). Data are presented as mean ± standard deviation. Statistical significance was set at *p* < 0.05.

## 3 Results

### 3.1 Pronounced pericyte degeneration and alterations in the diabetic human retina

**Figure 1:**
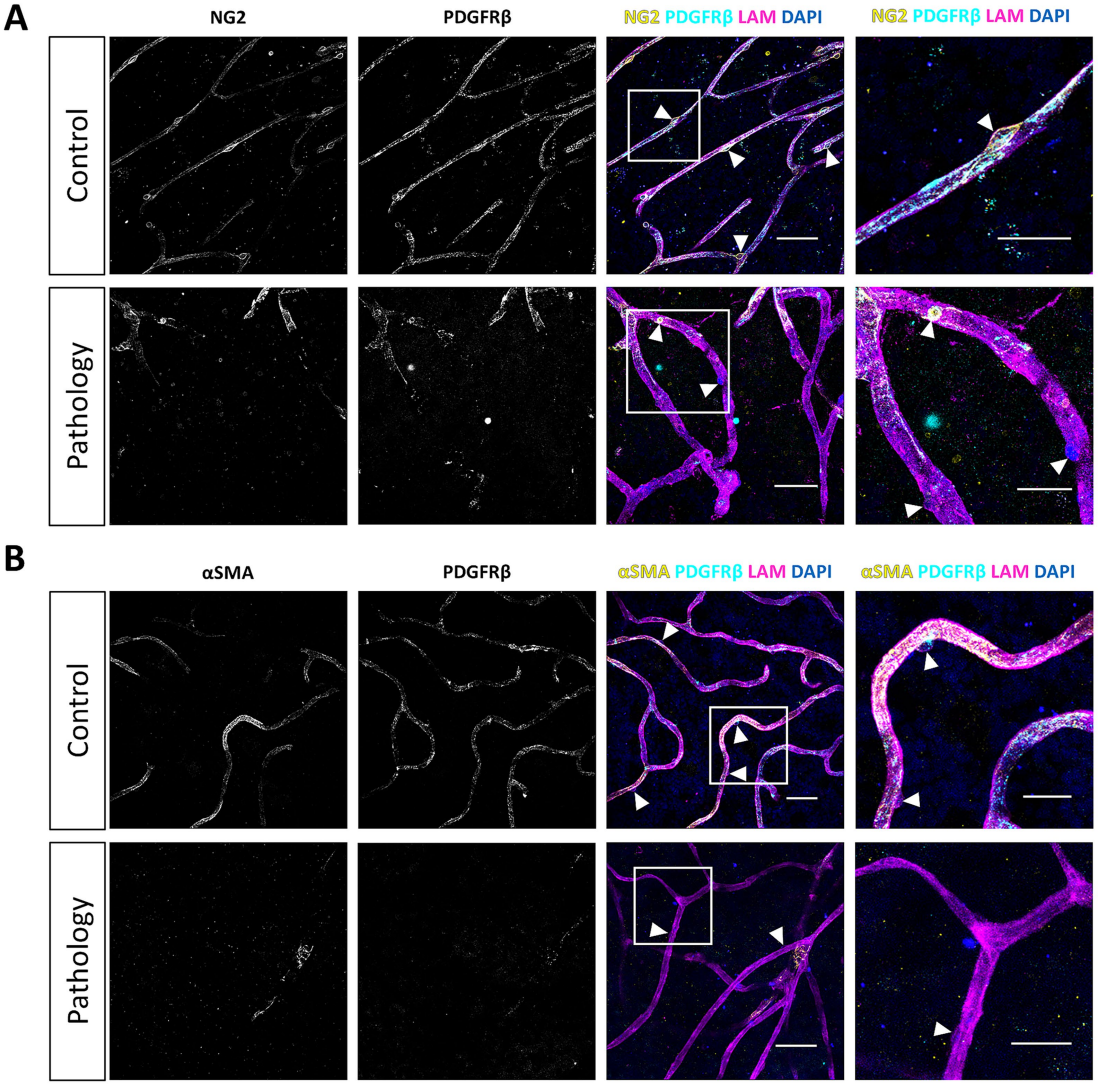
Loss of pericyte markers from capillaries in retina samples from donors with type 2 diabetes mellitus. Immunofluorescence 3D images of NG2 (yellow) and PDGFRβ (cyan) staining **(A)**, and of αSMA (yellow) and PDGFRβ (cyan) staining **(B)**. Images show expression in the human retina under physiological control conditions, and the loss of pericyte marker expression under pathological conditions. The basal lamina of blood vessels is stained for laminin (LAM, magenta), and nuclei are stained with DAPI (blue). White arrowheads indicate examples of pericytes. Scale bars: 50 μm. Images on the far right show higher magnification of the boxed region; scale bars: 25 µm.

**Figure 2:**
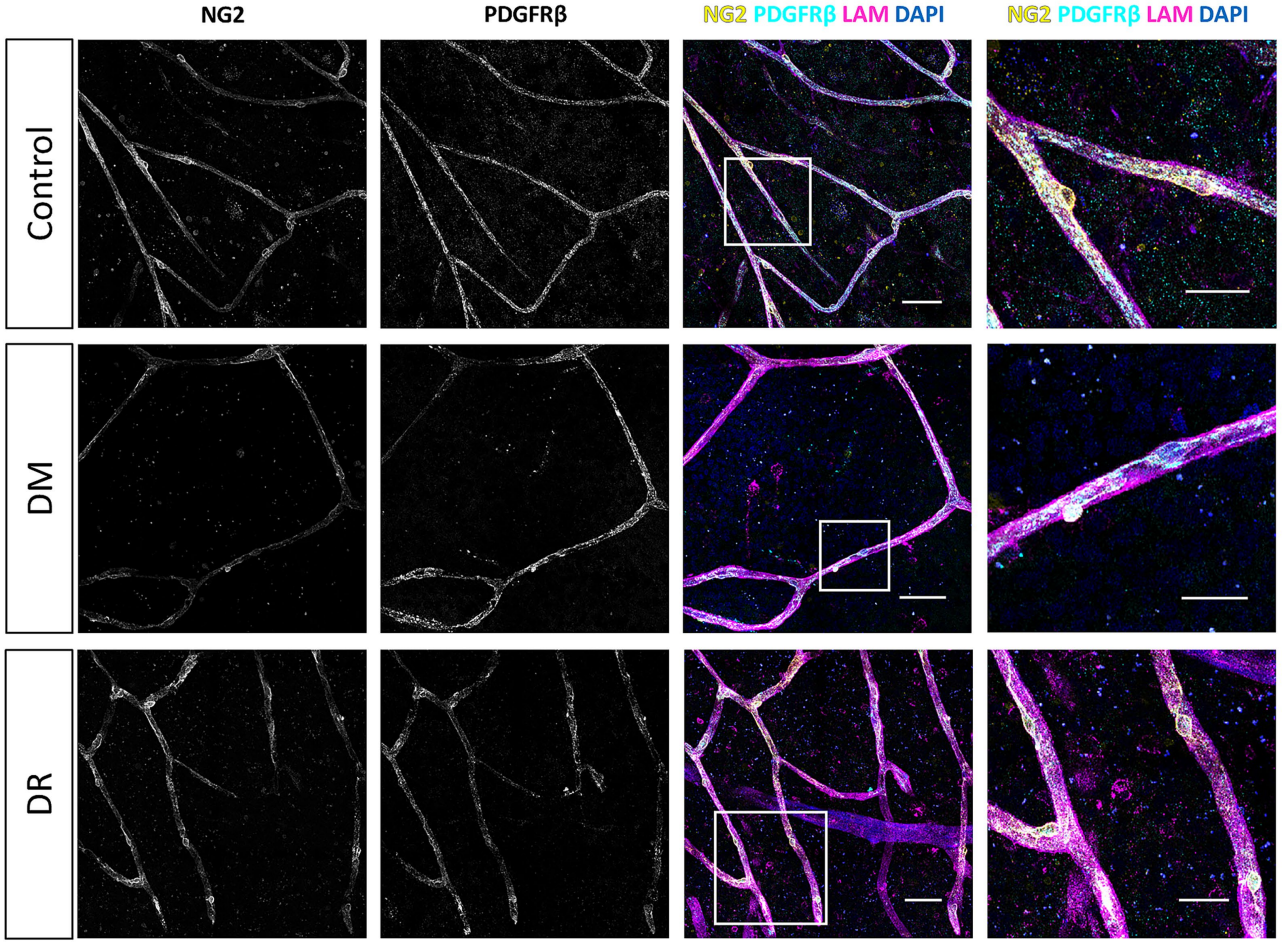
Immunofluorescence staining of NG2 and PDGFRβ in the human retina. Representative 3D confocal microscopy images of NG2 (yellow) and PDGFRβ (cyan) immunofluorescence staining on capillaries in retinal flatmounts from non-diabetic donors (control), donors with type 2 diabetes mellitus (DM), and donors with diabetic retinopathy (DR). The basal lamina of blood vessels is stained for laminin (LAM, magenta), and nuclei are stained with DAPI (blue). Scale bars: 50 μm. Images on the far right show higher magnification of the boxed region; scale bars: 25 μm.

NG2/PDGFRβ double immunofluorescence staining of retinal flatmounts yielded detailed comprehensive 3D reconstructions of the pericyte morphology (Figures 1A, 2). Confocal microscopy tile scans from each patient group revealed the pericyte distributions in all disease states, with fine morphological details captured by high-resolution imaging (FiglinQ-link-NG2). NG2 staining robustly labeled pericyte cell bodies, and distinctly outlined their processes along capillaries in all patient groups. NG2-positive pericytes with spherical somata were predominantly localized at the branching points of blood vessels. PDGFRβ expression exhibited an intracellular granular pattern, showing diffuse distribution across most of the vasculature in all patient groups. Overall, the NG2 and PDGFRβ signals showed substantial, although not complete, colocalization in the retinas of all patient groups. Notably, compared to control retinas, type 2 DM and DR retinas exhibited a higher prevalence of capillaries lacking expression of both markers.

**Figure 3:**
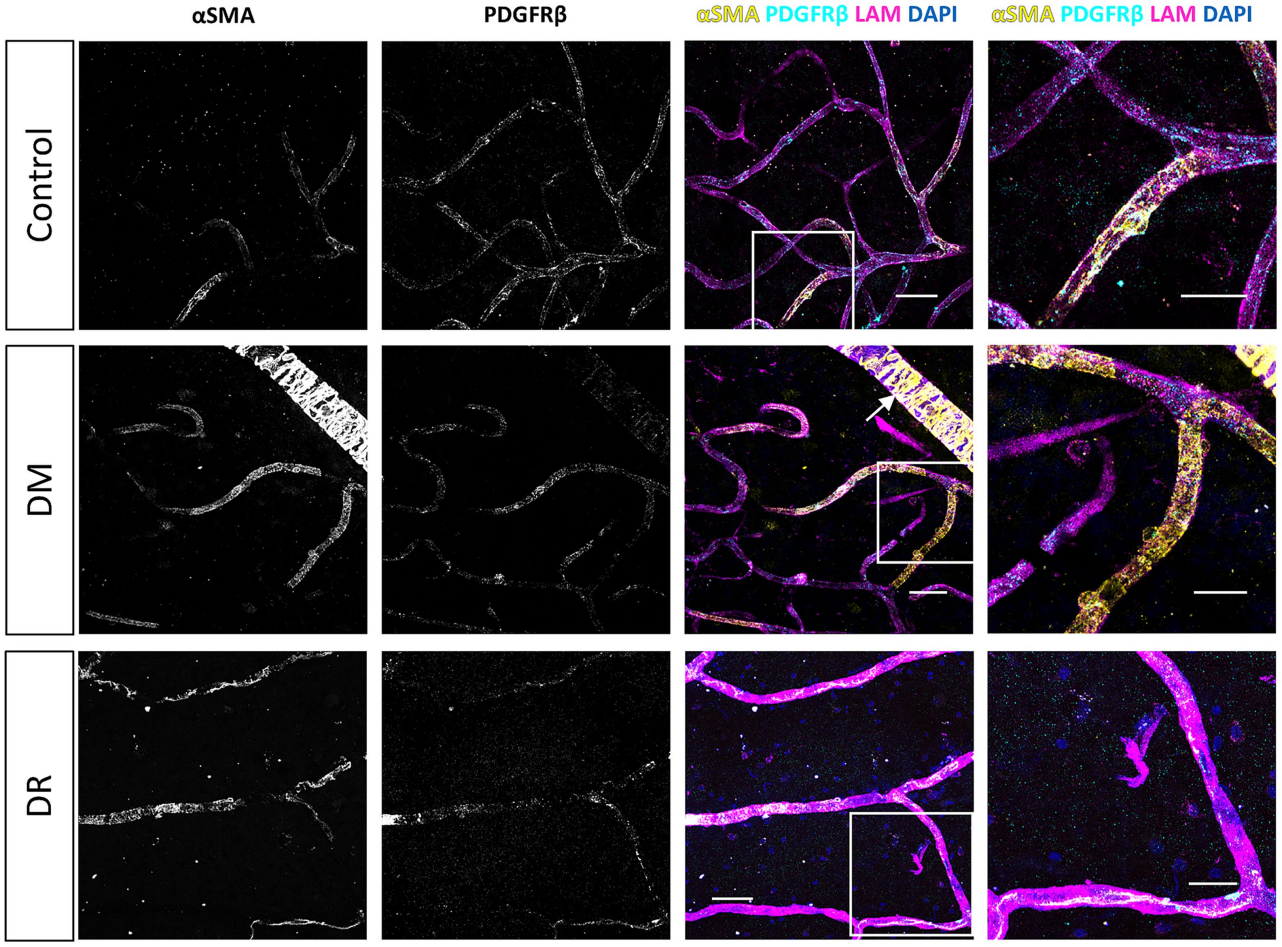
Immunofluorescence staining for αSMA and PDGFRβ in human retina samples. Representative 3D confocal microscopy images of αSMA (yellow) and PDGFRβ (cyan) immunofluorescence staining in retinal flatmounts from non-diabetic control donors (control), donors with type 2 diabetes mellitus (DM), and donors with diabetic retinopathy (DR). The basal lamina of blood vessels is stained for laminin (LAM, magenta), and nuclei are stained with DAPI (blue). Arrow indicates arteriole. Scale bars: 50 μm. Images on the far right show higher magnification of the boxed region; scale bars: 25 μm.

We also performed αSMA/PDGFRβ double immunofluorescence staining in human retinas from the same donors. Confocal microscopy tile scans of αSMA/PDGFRβ co-stained human retinal flatmounts were obtained from each patient group (FiglinQ-link-αSMA). In contrast to the ubiquitous NG2 and PDGFRβ expression, αSMA immunoreactivity was predominantly localized in arteriolar smooth muscle cells, with only segments of capillaries being positive for αSMA, across all patient groups (Figures 1B, 3). αSMA expression was consistently colocalized with PDGFRβ expression. Capillaries lacking both pericyte markers were observed in some regions in most DM and DR retinas, and in one control retina sample (Figure 1B). Retina samples from two DR donors exhibited reduced αSMA staining, compared to retina samples from both control and DM donors.

Both double staining experiments revealed that total PDGFRβ expression in the retinal vasculature was generally lower in DR donors, compared to both control and DM donors. Based on the presence or absence of NG2, PDGFRβ, and αSMA, we identified distinct pericyte subpopulations in retinal flatmounts from all donors. These subpopulations showed similar overall distributions across patient groups, except that pericytes positive for both PDGFRβ and αSMA were presented at lower proportions in the DM and DR groups, compared to controls.

Apart from pericyte-specific alterations, both control and DM retinas generally exhibited a well-organised vasculature. In contrast, immunofluorescence staining revealed regional vascular disorganization in all retina samples from DR donors, characterized by disrupted vessel alignment or, for some donors, the presence of microaneurysms (Supplementary Figure 3), which are hallmarks of DR (Little et al., 2022).

**Figure 4:**
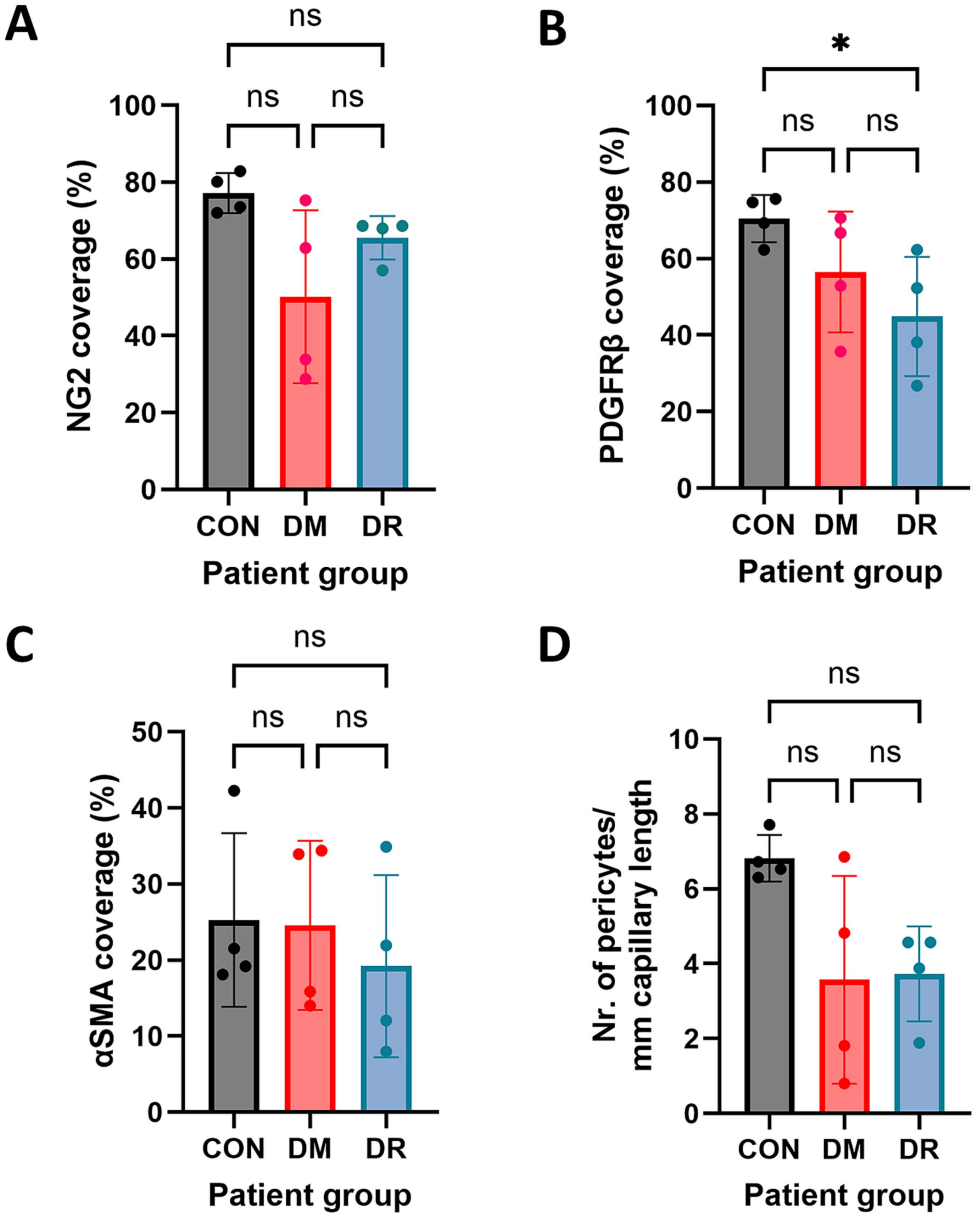
Quantification of retinal vascular staining coverage and pericyte cellular density, based on pericyte markers. Quantification of NG2 **(A)**, PDGFRβ **(B)**, and αSMA **(C)** vascular coverage in human retina samples from non-diabetic controls (CON), donors with type 2 diabetes mellitus (DM), and donors with diabetic retinopathy (DR) (*n* = 4). Quantification of NG2 pericyte cell number in the retina was normalized to laminin (LAM)-positive capillary length **(D)**. Each dot represents an individual donor. Data are expressed as the mean ± SD. *p < 0.05.

The immunofluorescence staining results were used to quantify the vascular staining coverage of these markers and the pericyte cellular density along retinal capillaries. Among non-diabetic controls, NG2-positive pericytes covered approximately 80% of retinal capillaries (Figure 4A), while PDGFRβ-positive pericytes covered approximately 70% (Figure 4B), indicating that pericytes covered nearly the entire abluminal surface area of retinal capillaries. Compared to controls, diabetic retinas, with and without DR, showed reduced vascular staining coverage based on NG2 expression, although substantial variability was observed among diabetic donors (Figure 4A). Additionally, PDGFRβ coverage was significantly reduced to 45% in DR retinas, compared to non-diabetic controls (*p =* 0.043) (Figure 4B). All groups showed consistently low vascular staining coverage of αSMA. Approximately 25% of capillaries displayed αSMA-positive pericytes, with no significant differences between groups (Figure 4C).

**Figure 5:**
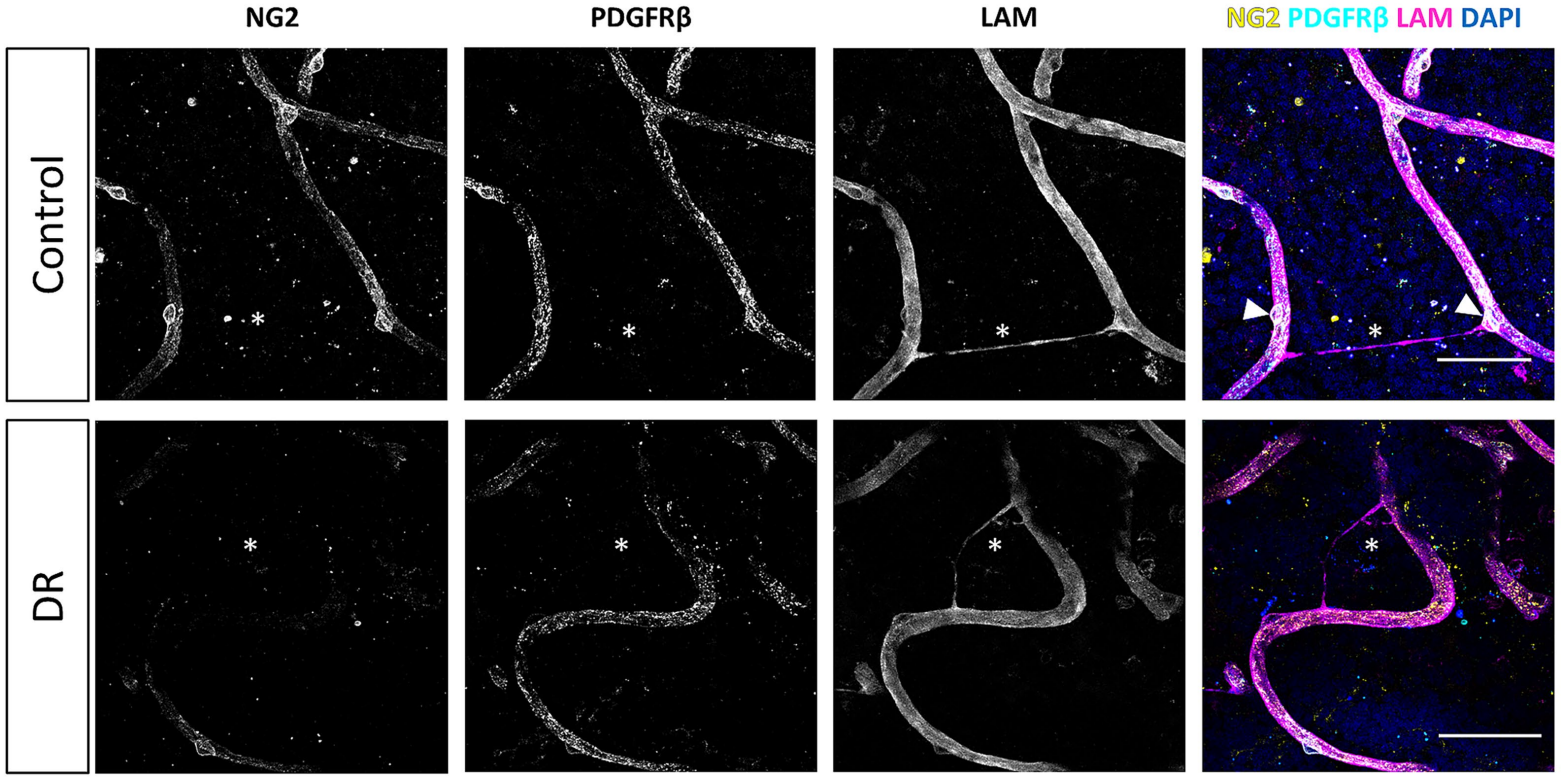
Basal lamina bridges in the human retina. Confocal microscopy images of basal lamina bridges in human retina samples from a non-diabetic (control) donor and a donor with diabetic retinopathy (DR). Laminin (LAM, magenta) staining reveals the basal lamina of the microvasculature in the retina, including the basal lamina bridges. Pericytes are stained for NG2 (cyan) and PDGFRβ (yellow), and nuclei are stained with DAPI (blue). White asterisks indicate basal lamina bridges. White arrowheads indicate pericyte somata in close proximity to a basal lamina bridge. Scale bars: 50 μm.

Pericyte density was calculated to be 7 pericytes/mm capillary length in the non-diabetic retinas, and was reduced to 4 pericytes/mm capillary length in both DM and DR retinas (Figure 4D). These results are in accordance with previous reports of early pericyte loss in DM, prior to DR onset (van der Wijk et al., 2018; Little et al., 2022). Across all groups, we observed thin laminin-positive tubular structures bridging capillaries that lacked pericyte marker expression (Figure 5), with a predominance among control donors.

In summary, quantitative immunofluorescence analysis revealed substantial reductions of NG2-positive pericyte density, and vascular NG2 and PDGFRβ staining coverage, in diabetic retinas, both with and without DR, compared to non-diabetic controls. We did not observe any DM-induced alterations in αSMA coverage. These findings correspond to the known pericyte alterations in DM and DR during early disease stages, preceding clinically detectable DR (van der Wijk et al., 2018; Little et al., 2022).

### 3.2 Subtle reduction in the vascular staining coverage of pericyte markers in DM and AD brain samples, with unchanged pericyte density

**Figure 6:**
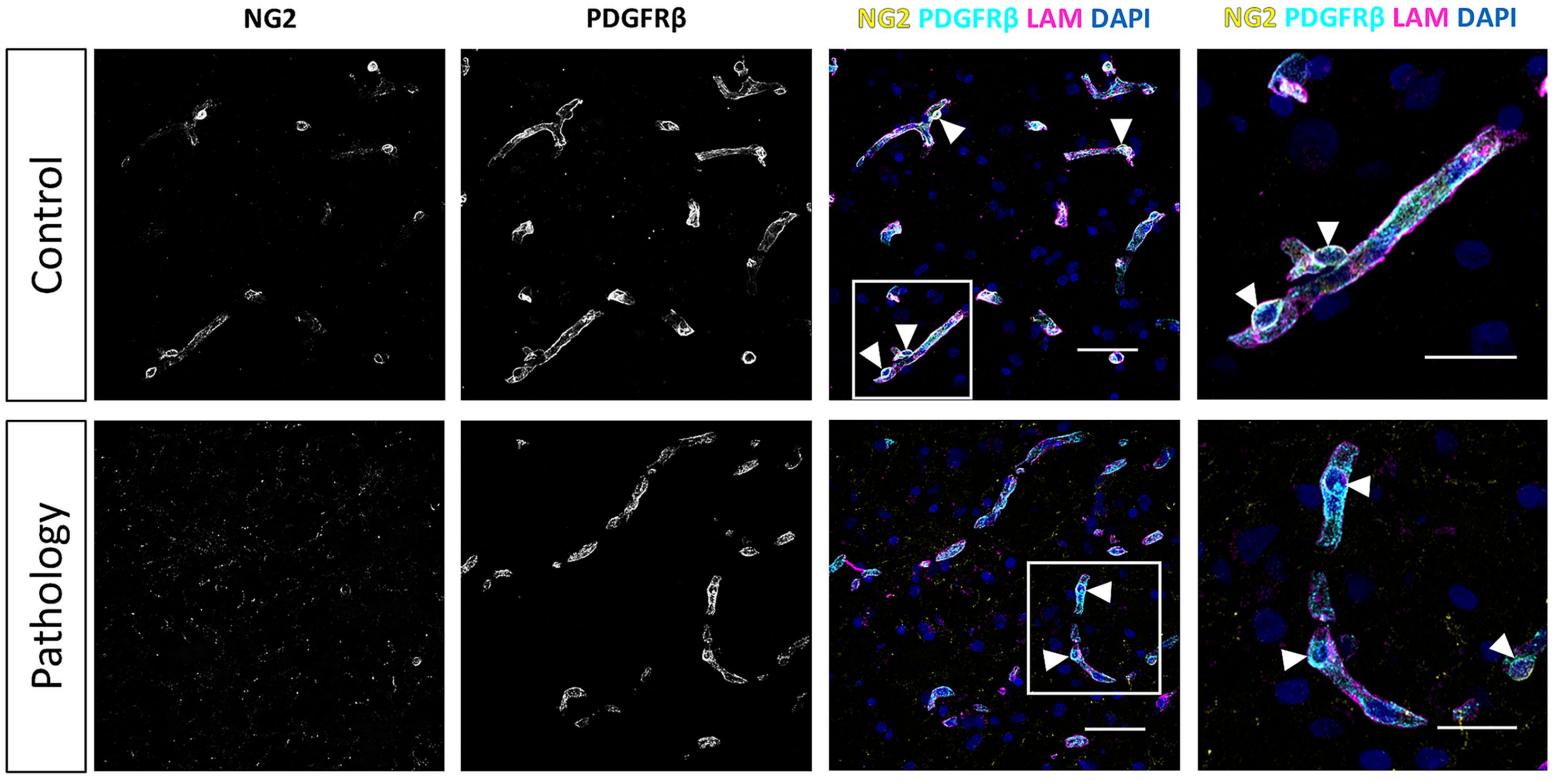
Immunofluorescence staining of NG2 and PDGFRβ in the human frontal cortex. Immunofluorescence images of NG2 (yellow) and PDGFRβ (cyan) staining in the human frontal cortex under physiological control conditions, and showing the loss of NG2 expression under pathological conditions. The basal lamina of blood vessels is stained for laminin (LAM, magenta) and nuclei are stained with DAPI (blue). White arrowheads indicate examples of pericytes. Scale bars: 50 μm. Images on the far right show higher magnification of the boxed region; scale bars: 25 μm.

**Figure 7:**
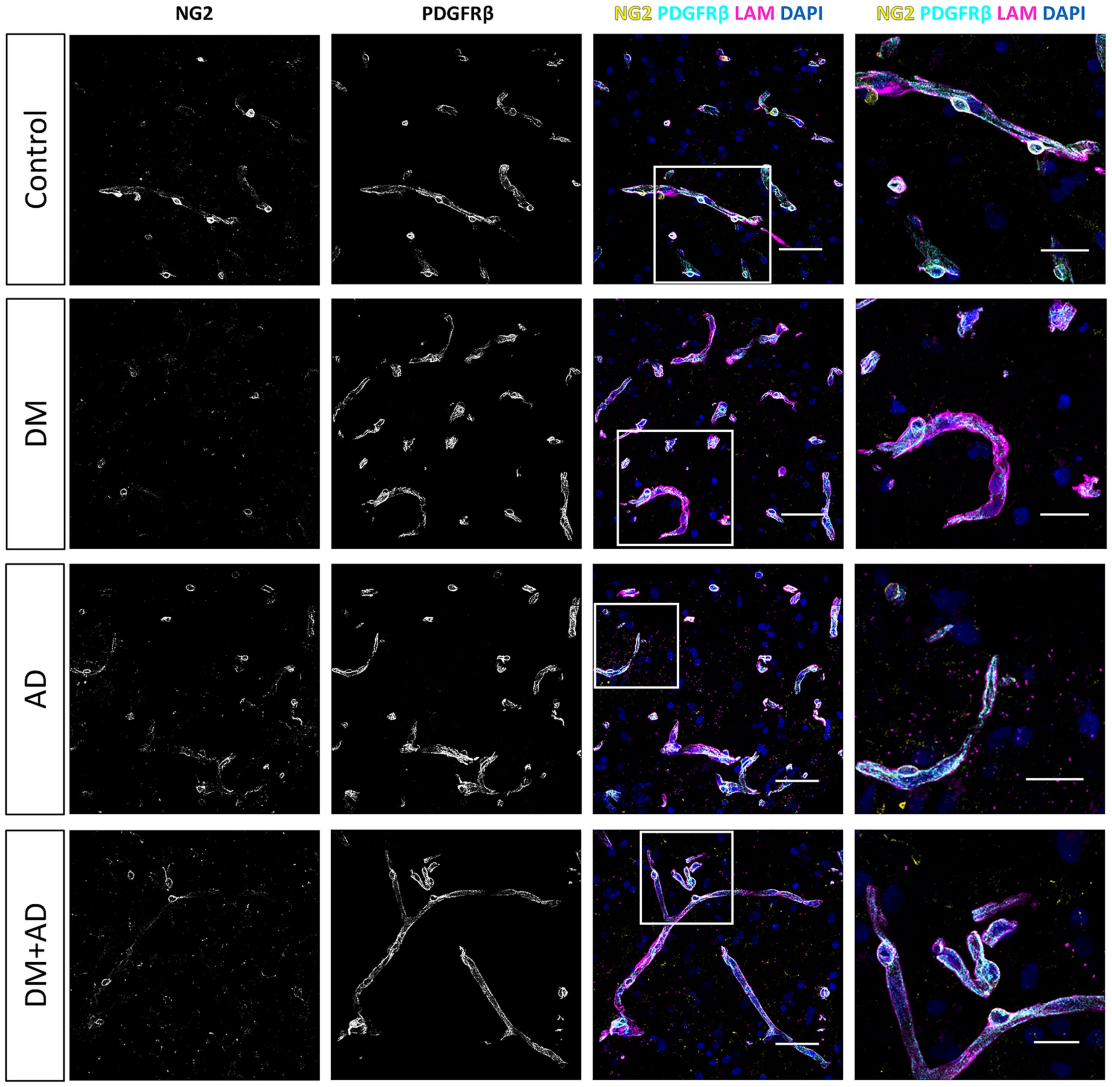
Immunofluorescence staining for NG2 and PDGFRβ in human brain samples from donors with diabetes mellitus (DM) and donors with Alzheimer’s disease (AD). Representative confocal microscopy images of NG2 (yellow) and PDGFRβ (cyan) immunofluorescence staining in human frontal cortex cryosections (10 μm) from control donors, donors with type 2 DM, donors with AD, and donors with both DM + AD. The basal lamina of blood vessels is stained for laminin (LAM, magenta), and nuclei are stained with DAPI (blue). Scale bars: 50 μm. Images on the far right show higher magnification of the boxed region; scale bars: 25 μm.

**Figure 8:**
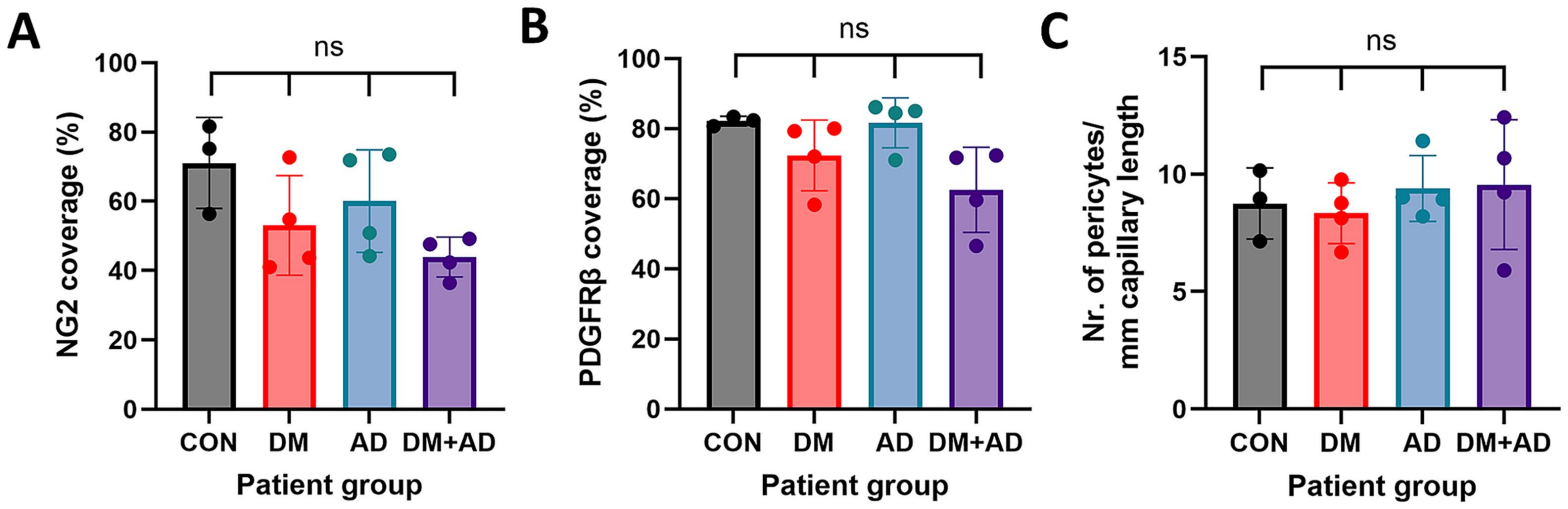
Quantification of cerebral vascular coverage, and pericyte density, based on pericyte markers. Quantification of NG2 **(A)** and PDGFRβ **(B)** vascular staining coverage in human frontal cortex samples from control donors (CON) (*n* = 3), donors with type 2 diabetes mellitus (DM) (*n* = 4), donors with Alzheimer’s disease (AD) (*n* = 4), and donors with both DM + AD (*n* = 4). Quantification of the NG2 pericyte cell number in the retina is normalized to CD31-positive capillary length **(C)**. Each dot represents an individual donor. Data are expressed as the mean ± SD.

Consistent with our observations in retina samples, NG2 expression in the frontal cortex was predominantly localized to pericyte cell bodies and, to a lesser extent, in the pericyte processes (Figures 6, 7). In contrast, PDGFRβ expression was diffusely and evenly distributed throughout the pericyte cell body and processes, exhibiting a very different pattern compared to the granular pattern observed in pericytes in the retina. We did not observe brain sample regions with complete loss of both pericyte markers in any patient group. Some areas contained capillaries stained for PDGFRβ but lacking NG2 expression (Figure 6). Additionally, lower NG2 expression was generally observed in the patient groups compared to controls (Figure 7). Quantitative analysis demonstrated that approximately 70–80% of cortical capillaries were covered by NG2-positive and PDGFRβ-positive pericytes in control brain samples, which was comparable to our observations in control retina samples (Figures 8A,B). Compared to controls, all patient groups (type 2 DM, AD, and type 2 DM + AD) exhibited reduced vascular staining coverage of NG2 (Figure 8A) and PDGFRβ (Figure 8B), with the DM + AD group exhibiting the lowest NG2 coverage (44%) and PDGFRβ coverage (63%). Quantitative analysis revealed that the pericyte density was modestly higher in control brain tissue (9/mm) compared to control retinal tissue (7/mm) (Figure 8C). However, pericyte density did not significantly differ among type 2 DM, AD, DM + AD, and control brain samples.

We did not observe αSMA staining in human brain samples using the same antibody and fixation method that were applied to the retina samples.

Overall, our comparative analysis revealed subtle reductions in the vascular staining coverage of pericyte markers in frontal cortex samples from donors with DM and AD, without alterations of pericyte cellular density.

## 4 Discussion

The aim of this study was to explore pericyte characteristics of the BBB in cases of type 2 DM and AD, with comparison to those of the iBRB in cases of type 2 DM. Our study is the first explorative quantitative analysis of this subject, and revealed an estimated vascular staining coverage of 70–80%, based on NG2 and PDGFRβ, under non-diabetic conditions. Compared to the human retina, the human brain samples exhibited similar widespread expression of NG2 and PDGFRβ staining in the vasculature, reflected by similar vascular staining coverage of 70–80%. Among the examined markers, NG2 and PDGFRβ showed the highest vascular coverage, closely approximating the vascular coverage and density of the pericyte population. In contrast, αSMA showed lower staining coverage of around 25% in the retina. Although previous studies have reported αSMA detection in cerebral microvasculature (Schlingemann et al., 1990; Bandopadhyay et al., 2001; Hutter-Schmid and Humpel, 2016), here we did not observe αSMA immunoreactivity in brain pericytes.

Our results revealed interesting trends in differential tissue-specific pericyte alterations between retina and brain tissue, related to pericyte cellular density in DM and AD. First, while both tissue types showed lower vascular staining coverage of pericyte markers in samples from donors with DM, quantitative assessment showed reduced pericyte cellular density only in retinas of diabetic patients. The unchanged cerebral pericyte cellular density may indicate that although reduced expression of pericyte markers leads to decreased vascular staining coverage, the marker expression in the pericyte somata remains detectable. Alternatively, pericyte somata may be preserved despite alterations of cell processes. Our study could not resolve these issues in the absence of electron microscopy. Secondly, among the brain samples, the DM + AD group showed marked reductions of both NG2 and PDGFRβ vascular staining coverage, which were not observed in the donor groups with only DM or only AD. This finding may indicate that pericyte alterations play a role via diabetes-specific mechanisms, specifically in patients with DM who develop AD. There remains a need for further research using human AD tissue, to better understand how diabetes-induced vascular pathology contributes to the development and progression of AD in DM.

The visualization of pericytes using marker-independent techniques, such as 3D electron microscopy (Abdelazim et al., 2022), offers superior resolution for defining pericyte morphology and spatial context; however, such approaches are often limited in human tissue studies by accessibility and practicality. Thus, here we employed immunofluorescence staining with commonly used pericyte markers, as a surrogate tool for assessing pericyte characteristics. These markers, including NG2, PDGFRβ, and αSMA, have been previously validated in retinal and brain tissues, through anatomical and morphological characterization and co-expression of pericyte markers (Armulik et al., 2011; Bakker et al., 2025). However, it must be noted that these markers are not entirely pericyte-specific, as they can also be expressed by other cell types. Moreover, their expression may not be uniform across all pericyte subpopulations or cellular compartments, potentially leading to underrepresentation. Despite their limitations, these markers remain the most valuable tools for approximating pericyte coverage and density in human tissue.

Our study is also subject to several other limitations. It was constrained by small sample sizes, due to the rarity of human brain and retina samples from donors with DM and/or AD. Additionally, there was high inter-patient variation, possibly due to comorbidities. Together, these limitations resulted in low statistical power. Therefore, the observed pathological differences should be regarded as trends, and may not reflect the true situation in the total patient population. There remains a need to increase the number of donors per patient group, to improve the statistical power. Another key limitation is the lack of matched eye and brain samples from the same donor, which prevents direct comparison of retinal and cerebral pathology. Furthermore, our analysis was confined to the frontal cortex, and pathological changes in AD vary according to region, e.g., with atrophy beginning in the hippocampus (Planche et al., 2022). Additionally, it is possible that pericyte cellular density was underestimated because pericytes with a flat morphology, or with somata located on the underside of blood vessels, can be difficult to detect in post-mortem tissue, in contrast to the easily identifiable pericytes with a nodular shape. Discrepancies between our results and those in the literature may be attributed to post-mortem artifacts in our study, partial pericyte marker expression, or interspecies differences. Notably, previous reports have detected αSMA in brain pericytes, while this was not the case in our present study. This specific discrepancy may arise from antibody or fixation limitations; reduced αSMA expression in our aged donor cohort, consistent with reported age-related reductions of 20–30% (Gyori et al., 2025); or the expression of alternative actin isoforms, such as smooth muscle *γ*-actin (Grant et al., 2019; Gyori et al., 2025).

Despite these limitations, the observed trends regarding differences in pericyte pathology between the retina and brain may indicate that pericytes are differently affected, or play a different role in pathology, in the NVU of the brain versus the retina, in DM alone, AD alone, and in DM patients developing AD. Alternatively, the brain may be more protected by compensating mechanisms (Bobkova and Vorobyov, 2015). Notably, the distinct and well-characterized early pericyte pathology observed in the retina in DM may explain why the retina shows microvascular damage in DM at an earlier stage, compared to the cerebral microvasculature (Little et al., 2022). The presently observed reduction in cerebral vascular staining coverage of pericyte markers aligns with previous findings in the diabetic rat brain (Liu et al., 2021) and in the post-mortem human AD brain (Sengillo et al., 2013; Halliday et al., 2016; Kirabali et al., 2020). On the other hand, the currently reported preserved pericyte density contrasts with previous studies, which have reported significant decreases (Sengillo et al., 2013; Halliday et al., 2016; Schultz et al., 2018; Ding et al., 2020; Hase et al., 2024) or increases (Fernandez-Klett et al., 2020; Ding et al., 2021) of pericyte density in the human AD brain. Pericyte dysfunction and loss compromise the integrity of the BBB and iBRB, potentially affecting neural tissue homeostasis in the brain and retina, respectively (Klaassen et al., 2013; Little et al., 2022). The contradictory findings regarding pericyte loss in brain capillaries in AD, in the literature together with our present findings, indicate that pericyte loss in the brain is not a consistent or universal feature of AD and DM. Alternatively, discrepancies may be explained by regional differences in cell bodies (Ding et al., 2021; Hase et al., 2024), or by differences among quantification approaches. Beyond tissue-specific differences in pericyte pathology, previous studies have identified additional tissue-specific variations, including regulation of the amyloidogenic and non-amyloidogenic pathways, neuronal tissue thinning, ganglion cell loss, tau protein accumulation, and alterations in microglial gene expression (den Haan et al., 2018; Bloomfield et al., 2024).

Our present findings, in a rare collection of human tissues, provide the first preliminary indication that there are distinct tissue-specific pathogenic mechanisms at play, which affect pericytes in the brain, as compared to the retina, in type 2 DM and AD. Moreover, our result suggest that retinal pericyte biomarkers may not be relevant as early predictors of cognitive impairment. Further work is needed to characterize these pericyte changes and other alterations in the BBB in type 2 DM and AD.

## Data Availability

The datasets presented in this study can be found in online repositories. The names of the repository/repositories and accession number(s) can be found at: https://create.figlinq.com/~iklaassen/141.
