# Supplementary material for: Differential pericyte pathology in the human retina and brain in diabetes mellitus and Alzheimer’s disease
Source: Front Neurosci. 2026 Feb 5;20:1749112. doi: 10.3389/fnins.2026.1749112 (PMC12916592; doi:10.3389/fnins.2026.1749112)
Supplement: Supplementary file 1 [file Supplementary_file_1.docx]

Supplementary Material

Macro for drawing blood vessel ROI

if (bitDepth() != 16) {

exit("This script requires a 16-bit image.");

}

setAutoThreshold("Default dark");

setOption("BlackBackground", false);

run("Analyze Particles...", "size=100-Infinity circularity=0.00-1.00 show=Nothing display clear add");

count = roiManager("count");

for (i = count-1; i >= 0; i--) {

roiManager("select", i);

diameter = getValue("Feret");

length = getValue("Major");

if (diameter < 10 || length > 20) {

roiManager("delete");

}

}

roiManager("Show All");

**Supplementary Figure 1**. ImageJ macro script 1 to automatically draw an ROI around blood vessels in immunofluorescence images.

Macro to set threshold for pericyte marker channel

threshold = 1.000;

setThreshold(threshold, 255);

setOption("BlackBackground", false);

run("Measure");

**Supplementary Figure 2.** ImageJ macro script 2 to remove background signal from immunofluorescence images using a pre-defined threshold.

**
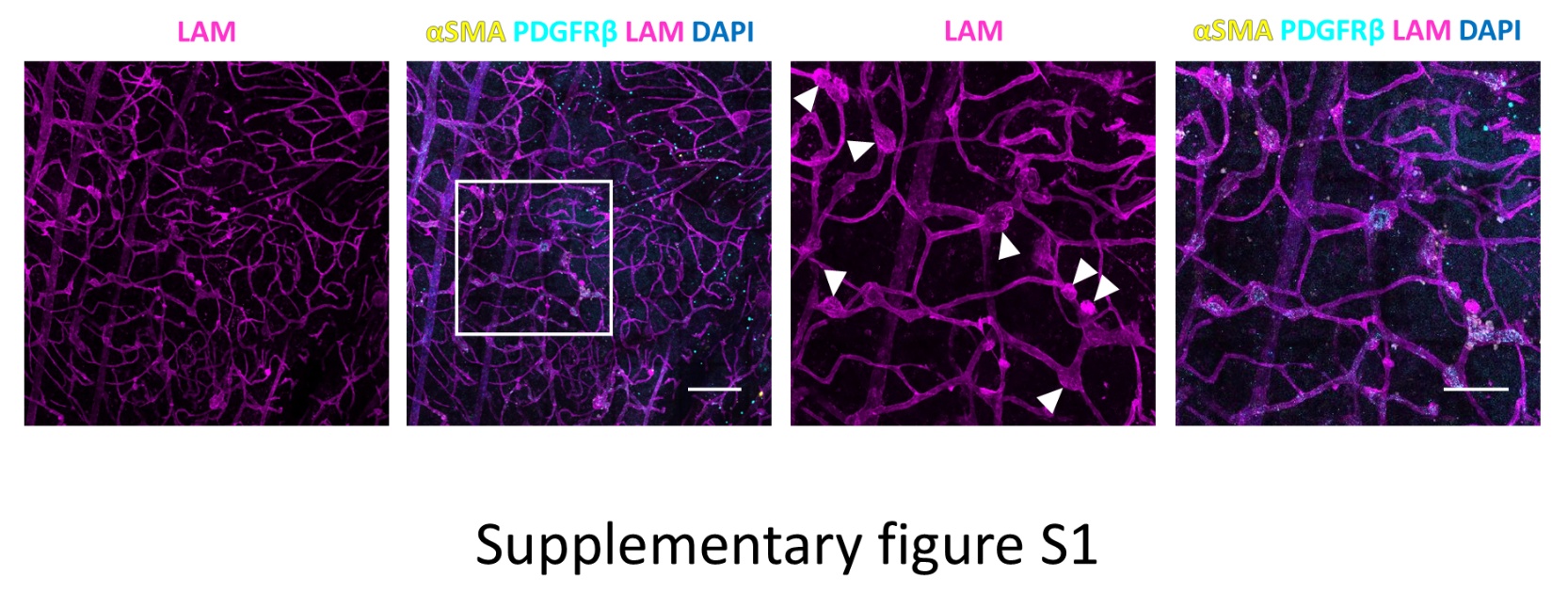
**

**Supplementary Figure 3.** Vascular disruptions in the human retinas from donors with diabetic retinopathy (DR). Immunofluorescence images of laminin (LAM, magenta), αSMA (yellow) and PDGFRβ (cyan) staining in the human DR retina. Nuclei are stained with DAPI (blue). White arrowheads indicate examples of microaneurysms. Scale bars: 200 µm. Images on the far right show higher magnification of the boxed region; scale bars: 100 µm.
